# Mitochondrial proteomic profiling reveals increased carbonic anhydrase II in aging and neurodegeneration

**DOI:** 10.18632/aging.101064

**Published:** 2016-10-10

**Authors:** Amelia Pollard, Freya Shephard, James Freed, Susan Liddell, Lisa Chakrabarti

**Affiliations:** ^1^ School of Veterinary Medicine and Science, University of Nottingham, Sutton Bonington, LE12 5RD, UK; ^2^ School of Biosciences, University of Nottingham, Sutton Bonington, LE12 5RD, UK

**Keywords:** mitochondria, ageing, skeletal muscle, brain, proteomics, carbonic anhydrase

## Abstract

Carbonic anhydrase inhibitors are used to treat glaucoma and cancers. Carbonic anhydrases perform a crucial role in the conversion of carbon dioxide and water into bicarbonate and protons. However, there is little information about carbonic anhydrase isoforms during the process of ageing. Mitochondrial dysfunction is implicit in ageing brain and muscle. We have interrogated isolated mitochondrial fractions from young adult and middle aged mouse brain and skeletal muscle. We find an increase of tissue specific carbonic anhydrases in mitochondria from middle-aged brain and skeletal muscle. Mitochondrial carbonic anhydrase II was measured in the Purkinje cell degeneration (*pcd^5J^*) mouse model. In *pcd^5J^* we find mitochondrial carbonic anhydrase II is also elevated in brain from young adults undergoing a process of neurodegeneration. We show *C.elegans* exposed to carbonic anhydrase II have a dose related shorter lifespan suggesting that high CAII levels are in themselves life limiting. We show for the first time that the mitochondrial content of brain and skeletal tissue are exposed to significantly higher levels of active carbonic anhydrases as early as in middle-age. Carbonic anhydrases associated with mitochondria could be targeted to specifically modulate age related impairments and disease.

## INTRODUCTION

Brain ageing is associated with cognitive decline and neurodegeneration. Normal ageing often leads to levels of decline in cognition, with estimates of a fifth of people over 71 affected by impairment that is not classed as dementia [1]. Loss of mitochondrial functionality is implicated as a key factor leading to age related decline and the development of many neuro-degenerative diseases. Increasing our understanding of the changes that occur in the normal process of ageing is crucial to help distinguish between the biological features of disease and that of ageing itself. Delineating the expected changes within the lifetime of a mammal provides entry points to examine endogenous protective and degenerative pathways, these lend themselves as biomarkers or can present novel treatment targets.

Ageing research has focussed on the extension of lifespan. The mitochondrial free-radical theory of ageing suggests that reactive oxygen species (ROS) produced by the mitochondria cause a decline of molecular function, resulting in ageing [2]. Critically, the balance of beneficial and deleterious effects of mitochondrial ROS within a tissue during a lifetime still needs to be determined [3]. Recent evidence supporting a role for ROS in ageing found that increasing NADPH, by upregulation of the enzyme glucose-6-phosphate dehydrogenase (G6PD) in mice increased antioxidant defences which delayed ageing [4]. Mitochondrial dysfunction is a common feature of ageing and neurodegeneration and certain mitochondrial proteins have been shown to undergo oxidative damage in both of these processes [5–7]. However, ROS are unlikely to be the only factors contributing to age-related mito-chondrial dysfunction. An understanding of how mito-chondrial composition changes with age can shed light on the mechanisms affecting these organelles throughout the lifetime of mammals. A complication, when looking at diseases of ageing, is the inability to separate the effect of normal ageing from disease-related changes in the mitochondrion, for example in Parkinson's disease where mitochondrial changes are clearly important, yet not always specifically distinguishable from the effects of ageing [5, 8].

Proteomics targeted specifically at the mitochondrial fraction of the cell is a powerful approach to identify the expected age-related changes in tissues. A few studies have been reported where mitochondrial proteins have been examined in normal brain tissue [9, 10]. Existing studies of skeletal muscle senescence suggest that mitochondrial enzymes are largely increased in abundance, though how this information fits with decreased complex I activity in ageing mitochondria has not yet been determined [11].

Our study stems from an interest in mitochondrial proteins within tissues with a high capacity for oxidative phosphorylation. Both the brain and skeletal muscle undergo a degree of decline in advanced age. We profiled proteins of mitochondrial fractions isolated from young (~8weeks) and old (78 weeks) mouse brain and skeletal muscle. It is pertinent to point out that our samples were deliberately chosen to reflect youth versus middle-age, rather than true old-age (old mouse would be >104 weeks) [12]. Our intention was to maximise the possibility of identifying early changes that may be occurring prior to detectable functional losses rather than the ‘gravestones’ heralding end stage dysfunction. While it is illuminating to interrogate the proteome of a particular biological entity, neither of these are closed systems. Our study highlights the importance of making assessments across these groupings. We show that the pharmaceutical target carbonic anhydrase II is increased with age in mitochondria. To investigate the potential importance of changing levels of these proteins we looked to see whether carbonic anhydrases are also changed in a similar manner in the Purkinje Cell Degeneration (*pcd^5J^*) mouse model of neuro-degeneration. The *pcd^5J^* mouse is an excellent model to study the effect of a pure mitochondrial neuro-degenerative phenotype that occurs early in life [13–15, 7]. We compared our findings in the neuronal and non-neuronal tissues with what we found in *pcd^5J^* to understand whether the levels of carbonic anhydrase found are likely to be a protective or dysfunctional alteration. We now are able to provide the molecular context of normal mitochondrial ageing which needs to be fully considered as carbonic anhydrase inhibitor therapy becomes more widely applied in diseases affecting our ageing populations.

## RESULTS AND DISCUSSION

### The mitochondrial proteome is different in young and old murine skeletal muscle tissue

**Figure 1:**
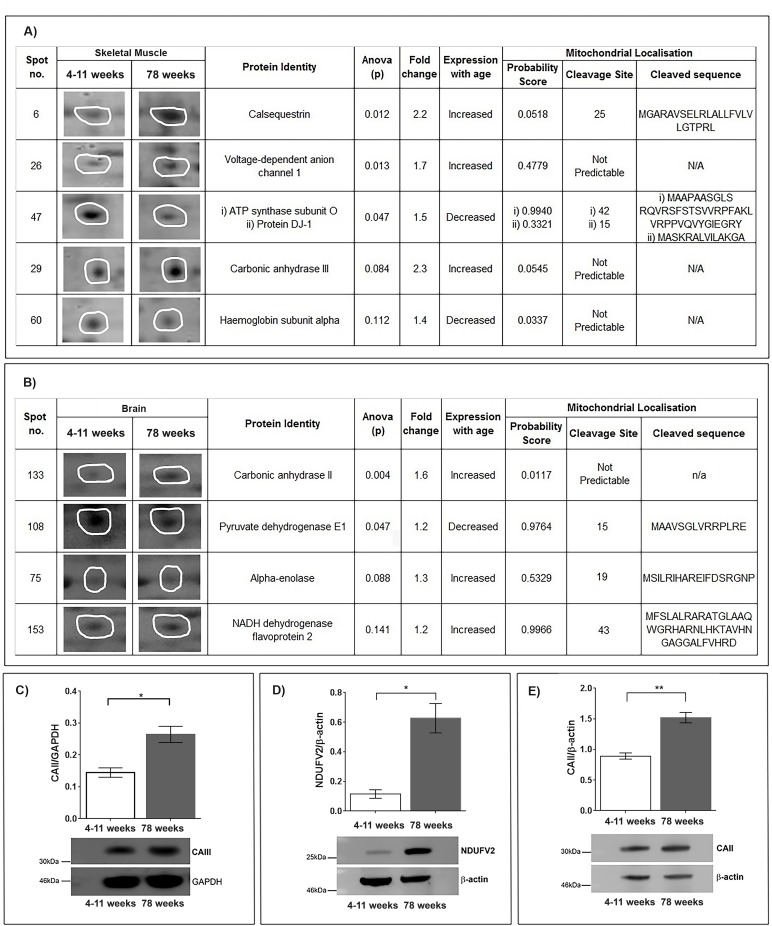
(**A**) Identification of protein changes with age in the skeletal muscle mitochondrial proteome. Five protein spots were selected after (SameSpots) analysis comparing murine skeletal muscle mitochondria aged 4-11 weeks (n=5) and 78 weeks (n=5). Representative protein spot images, statistical analyses (one-way ANOVA) and identities of the proteins MASCOT) are shown. Mitochondrial localisation probability was calculated (Mitoprot) and is shown along with predicted cleavage sites and sequence. (**B**) Identification of proteins that change with age in the skeletal muscle mitochondrial proteome. Four protein spots were selected after (SameSpots) analysis comparing murine brain mitochondria aged 4-11 weeks (young) (n=3) and 78 weeks (old) (n=3). Representative protein spot images, statistical analyses (one-way ANOVA) and identities of the proteins (MASCOT) are shown. Mitochondrial localisation probability was calculated (Mitoprot) and is shown along with predicted cleavage sites and sequence. (**C**) Carbonic anhydrase III protein levels increase in aged skeletal muscle mitochondria. Carbonic anhydrase III, normalised to GAPDH, is significantly increased in 78 week old (n=4) skeletal muscle mitochondria compared with 4-11 week old (n=4) skeletal muscle mitochondria, p=0.0105. (**D**) NADH dehydrogenase flavoprotein 2 protein levels increase in aged brain mitochondria. NADH dehydrogenase flavoprotein 2, normalised to beta-actin, is significantly increased in 78 week old (n=4) brain mitochondria compared to 4-11 week old (n=4) brain mitochondria, p=0.0109. (**E**) Carbonic anhydrase II protein levels increase in aged brain mitochondria. Carbonic anhydrase II, normalised to beta-actin, is significantly increased in 78 week old (n=4) brain mitochondria compared to 4-11 week old (n=4) brain mitochondria *p*=0.0015. Columns display mean activity ± SEM. * = p<0.05 and **= p<0.03 two-tailed unpaired t-test with Welch's correction.

Six proteins were selected to have changed when comparing the young (4-11 week) and old (78 week) skeletal muscle mitochondrial proteomic profiles (Figure 1A). Carbonic anhydrase III (discussed later), calsequestrin and Voltage Dependent Anion Channel 1(VDAC1) increase with age in the old skeletal muscle mitochondria (*p*<0.05). Calsequestrin increases with a greater than two-fold change between the young and older mitochondria. It has recently been shown that loss of calsequestrin leads to mitochondrial dysfunction and oxidative stress in skeletal muscle [16]. It could be interpreted therefore that an upregulation of calse-questrin in this case is a protective response rather than being reflective of muscle decline; a study in postmenopausal women also identifies a (smaller fold) increase in total skeletal muscle calsequestrin [17]. Overexpression of calsequestrin in cardiomyocytes suggests that endoplasmic reticulum calcium stores may be enhanced perinuclearly to provide an independent compensatory effect in the case of misregulated calcium homeostasis. Mitochondria also accumulate peri-nuclearly and are regulators of calcium signalling providing more evidence that the upregulation in calsequestrin that we observe is protective in ageing skeletal muscle [18]. In skeletal muscle mitochondria we can confirm definite changes in haemoglobin subunit alpha, ATP synthase and VDAC1. These mitochondrial proteins have previously been shown to be differentially regulated in ageing and our data confirm that these are likely important regulators of ageing in skeletal muscle mitochondria [5, 19, 20].

### The mitochondrial proteome is also distinctly different between young and old murine brain tissue

In brain mitochondrial fractions our top list defines pyruvate dehydrogenase E1, alpha enolase and NADH flavoprotein 2 as changed between the young (4-11 weeks) and old (78 weeks) brain mitochondrial proteome (Figure 1B). Pyruvate dehydrogenase is known to decline through the brain with age and enolase has recently found to be decreased on the CD4(+) T cell surface in a small study of older males [21]. However, this is the first time these have been shown to be changed in association with ageing of the mitochondrial organelle. NADH dehydro-genase flavoprotein 2 (NDUFV2) a complex 1 protein is confirmed to be increased in the old mitochondria (*p*<0.005), agreeing with the perceived increase in complex 1 enzymes reported in senescent muscle [11] (Figure 1D). Mutations and variation in NDUFV2 are associated with disorders of the brain and ageing [22–25]. Our finding could suggest that the variations and mutations have a subtle effect on NDUFV2, which is most detrimental when upregulated, perhaps for neuroprotection in middle-age.

### Carbonic anhydrase II and III are significantly increased in mitochondria isolated from older mice

Interestingly we show that two isoforms of carbonic anhydrase (CAII and CAIII) increase in both 78 week old brain mitochondria and 78 week old skeletal muscle mitochondria, respectively. Carbonic anhydrases are zinc metalloenzymes that catalyse the reversible hydration of carbon dioxide to bicarbonate. Carbonic anhydrase II binds to Na+/H+ exchanger altering pH [26]. Carbonic anhydrases also catalyse the reversible hydration of CO^2^, HCO_3_^−^ and H^+^ [27]. Therefore, the role of carbonic anhydrases in maintaining the pH environment of the cell and specifically the mitochondrion is important to delineate.

CAIII is the muscle-specific isoenzyme [28] whilst CAII is located in the cytosol and widely expressed in most tissues [29]. In our study carbonic anhydrase III is significantly increased in old skeletal muscle mitochondria compared with young muscle mitochondria *p*<0.005 (t-test) (Figure 1C). We also see that CAII is significantly increased in mitochondria isolated from old brain tissue *p*<0.05 (t-Test) (Figure 1E). Calculated probability scores suggest that CAII and CAIII are not predicted to cleave into mitochondrial targeted forms. It is possible that these proteins associate very tightly with the mitochondrion without necessarily entering the organellar space [30, 31]. Carbonic anhydrase III is not essential for survival in mouse and has been known for some time to increase in muscle with ageing and contraction [28, 32, 35]. A role in mitochondrial function has not yet been postulated for it, though we clearly observe a significant increase in the quantity of this protein in aged-muscle mitochondrial fractions.

CAII, belongs to the group of these isoenzymes that are pharmacologically targeted by inhibitors (such as acetazolamide) to treat a variety of disorders including glaucoma, cancer osteoporosis, epilepsy, neuro-psychiatric disorders and acute mountain sickness [36–39]. Methazolamide also a carbonic anhydrase inhibitor, has also been shown recently to prevent amyloid-beta induced mitochondrial dysfunction and is neuroprotective in mouse models of Alzheimer's disease [40].

### The esterase activity of carbonic anhydrase II is increased in the mitochondrial fraction of brain tissue from older animals

**Figure 2:**
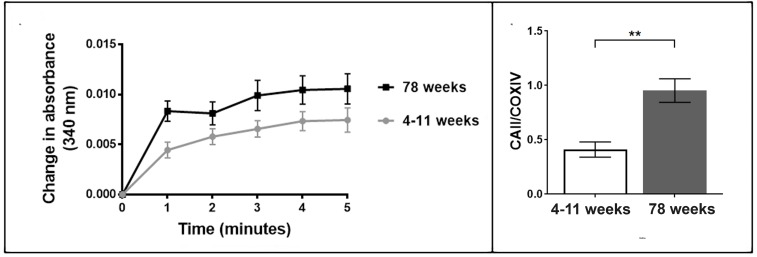
(**A**) Enzymatic activity of carbonic anhydrase II increases in aged brain mitochondria. The enzymatic activity of carbonic anhydrase II is higher in the 78 week old brain mitochondria compared to the 4-11 week brain mitochondria (three mitochondrial samples from individual animals in each age group and three technical replicates of each sample). Each mitochondrial sample contained 30 μg/μl of protein. (**B**) Carbonic anhydrase II protein levels increase in aged retina mitochondria. The protein levels of carbonic anhydrase II were measured in the murine retina mitochondria aged 4-11 weeks and 78 weeks. Carbonic anhydrase II significantly increases in retina mitochondria from older mice, p=0.028 (two-tailed unpaired t-test with Welch's correction). Replicates were obtained from individual animals (young n=6, old n=6). Columns display mean activity ± SEM. **= *p*<0.03 two-tailed unpaired t-test with Welch's correction.

We tested whether an upregulation of CAII protein corresponded with an increase in enzymatic activity. We measured the esterase activity of carbonic anhydrase II by monitoring the hydrolysis of 4-nitrophenyl acetate to form 4-nitrophenol. Mitochondria from aged brain tissue exhibited a higher rate of change in absorbance in comparison to mitochondria from young brain tissue throughout the 5-minute assay (Figure 2A). The rate of change at 1, 2 and 4 minutes were significantly higher in mitochondria from the old brain tissue (t-test) p<0.05. Our data show that the expression of CAII increases with ageing and this can be measured by the activity of CAII which is greater per mitochondrial unit in old versus young brain mitochondrial fractions.

### Carbonic anhydrase II protein levels in retina mitochondria significantly increase in aged animals

Advanced age leads to an increased risk of developing neurodegenerative diseases. Glaucoma is a neuro-degenerative disease that has been associated with oxidative stress and age related mitochondrial dys-function [41, 42]. We investigated the protein levels of CAII in retina mitochondria from aged mice (78 weeks). We observe a large increase in CAII in the retina mitochondria from aged mice in comparison to young mice (4-11 weeks), p<0.05 (Figure 2B). Our data indicate that CAII protein levels also increase in the retina with age.

### Carbonic anhydrase II protein levels significantly increase in brain mitochondria from the neurodegenerative mouse model *pcd*^5J^

**Figure 3:**
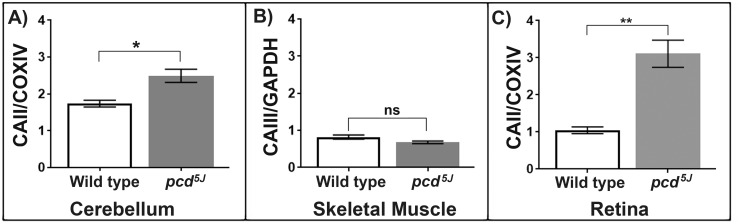
(**A**) Carbonic anhydrase II protein levels increase in the neurodegenerative mouse model, *pcd^5J^* cerebellum mitochondria. Carbonic anhydrase II, normalised to COXIV, is significantly increased in *pcd^5J^* cerebellum mitochondria compared to wild type animals aged (10-13 weeks old). Replicates were obtained from individual animals (wild type n=4, *pcd^5J^* n=4). (**B**) Carbonic anhydrase III protein levels are not significantly different between the *pcd^5J^* and wild type skeletal muscle mitochondria. CAIII protein levels, normalised to GAPDH, were compared between wild type (n=3) and *pcd*^5J^ (n=3) mice aged 10-13 weeks. Replicates were obtained from individual animals. (**C**) Carbonic anhydrase II accumulates in the *pcd^5J^* retina mitochondria. Carbonic anhydrase II levels are significantly higher in *pcd*^5J^ mice compared to wild type animals, *p*=0.0019. Replicates were obtained from individual animals (wild type n=6, *pcd*^5J^ n=6), all animals were between 9-17 weeks old. Columns display mean activity ± SEM. * = p<0.05, **= p<0.03 two-tailed unpaired *t*-test with Welch's correction.

The Purkinje Cell Degeneration mouse, *pcd*, is an autosomal recessive mutant and a model of neuro-degeneration. The *pcd*^5J^ mouse model has a mutation in the Nna1 gene that encodes a protein that is localised in the mitochondrion. Initially the *pcd*^5J^ mice are born with normal development of Purkinje cells but after 15 days rapid degeneration of the Purkinje cells occur, with over a 99% loss of Purkinje cells by around 3 weeks of age [13]. We used the *pcd^5J^* mouse model to investigate whether the changes in CAII levels are specific to the ageing process or are also a sign of neurodegeneration. Mitochondria were isolated from the cerebellum of *pcd*^5J^ and aged matched wild type animals (10-13 weeks). CAII protein levels are significantly elevated in the *pcd*^5J^ cerebellum mitochondria compared to wild type mitochondria, p<0.05 (Figure 3A). We propose that the increase in CAII in the brain with ageing is an early symptom of neurodegenerative decline.

### Carbonic anhydrase III protein levels are not significantly different between *pcd^5J^* and wild type skeletal muscle mitochondria

Skeletal muscle is unaffected by the loss of Nna1 function in *pcd^5J^* animals. The muscle specific isoform of carbonic anhydrase CAIII was found to be altered in the ageing skeletal muscle mitochondria. We wanted to test whether CAIII protein levels changed in the *pcd^5J^* mouse muscle to see whether the changes in CAII in neural tissues were indeed specific for ageing and neurodegeneration or whether the *pcd^5J^* mutant has a systemic alteration in carbonic anhydrases, even in unaffected tissues. We compared *pcd^5J^* skeletal muscle from the young mice that already showed signs of neurodegeneration in retina and cerebellum with age-matched wild type animals (10-13 weeks). We found that the quantity of CAIII is not significantly different in the *pcd^5J^* and wild type animals (Figure 3B). Since the *pcd^5J^* animals have functionally healthy skeletal muscle it is unsurprising that there is no significant difference between the quantity of CAIII in the wild type and mutant animals. However, this suggests very strongly that the alterations in carbonic anhydrases found in ageing and neurodegeneration are a harbinger of the dysfunction which ensues.

### The neurodegenerative mouse model, *pcd*^5J^, also shows significantly increased carbonic anhydrase II protein levels in retinal mitochondria

Retinal degeneration is a feature of the *pcd^5J^* animals and so we used this mouse model to investigate whether CAII increases in mitochondria in a disease state. We show CAII protein levels significantly increase in retina from *pcd^5J^* mice compared to aged matched wild type mice (2-4 weeks old), *p*<0.05 (Figure 3C). Our data suggest that the accumulation of CAII in retina mitochondria occurs during the normal ageing process and that increasing levels of CAII is also a feature of retinal degeneration. The CAII inhibitor dorzolamide hydrochloride is commonly used in the treatment of glaucoma to improve ocular perfusion. Dorzolamide hydrochloride has also been suggested to act as antioxidant, exerting its effect through intact mitochondria [43]. We now suggest that the action of dorzolamide hydrochloride in glaucoma should be analysed for its likely effect on the raised CAII levels we find in ageing retinal mitochondria.

### Increased carbonic anhydrase II reduces lifespan

**Figure 4:**
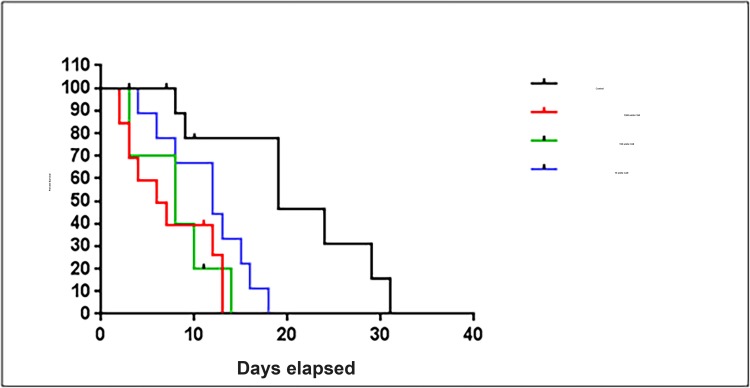
Exposure to carbonic anhydrase II treatment significantly shortens lifespan of *C.elegans. C. elegans* (strain CB5600) exposed to carbonic anhydrase II were recorded by Kaplan Meier survival plot (Log‐rank test, p=0.0006). Three concentrations of carbonic anhydrase II 1500 units, 150 units and 15 units were used (10 animals in each condition).

The six alpha-carbonic anhydrase isoforms 1 to 6 are encoded for in the *Caenorhabditis elegans* genome (cah-1, cah-2, cah-3, cah-4, cah-5 and cah-6) [44]. Wormbase searches revealed that the murine (*Mus musculus)* CAII gene has similar homology to the *C.elegans* gene cah-3. Both cah-3 and cah-4 in *C.elegans* are orthologs of the murine carbonic anhydrase II gene [45]. Ensemble searches showed that the amino acid sequence for the murine carbonic anhydrase II protein has sequence homology with four carbonic anhydrase proteins in *C.elegans*, cah-3, cah-5, cah-1 and cah-2. We tested whether the increase in CAII is a protective mechanism or a sign of dysfunction by exploring the effect of CAII on *C.elegans*. *C. elegans* (strain CB5600) were treated with three different concentrations of CAII (1500 units, 150 units and 15 units). Animals treated with CAII have a significant reduction in lifespan p=0.0006 (Log-rank test). The animals show a dose dependant response to increased levels of CAII (Figure 4). The median life-spans were 6, 8 and 12 days for animals treated with 1500 units, 150 units, 15 units respectively. In the same experiments control animals had a median lifespan of 19 days. The absolute maximum lifespans were 13, 14 and 18 days for animals treated with 1500 units, 150 units and 15 units respectively and the control animals had an absolute maximum lifespan of 31 days. Animals treated with the highest concentration of CAII (1500 units) had a 58% reduction in lifespan compared to the control animals, whilst animals treated with 150 and 15 units showed a reduction in lifespan by 55% and 42% respectively compared to the controls. Based on these findings we suggest that carbonic anhydrase inhibitors could be targeting the effects of mitochondrial ageing in neurons by reducing carbonic anhydrase levels to physiologically more youthful levels. CAII is likely to be an important regulator of the ageing process.

## CONCLUSIONS

We present a picture of complex proteomic profile changes in mitochondrial fractions with ageing. In particular, we observe accumulations of carbonic anhydrase isoenzymes with increased age. CAII protein levels were also found to increase in the cerebellum and retina mitochondria of the neurodegenerative disease mouse model, *pcd^5J^*. We propose that increased quantities of CAII play a detrimental role in the ageing process. Therapeutic use of carbonic anhydrase inhibitors are likely to be exerting an effect on mitochondrial populations and may be offering protection through maintenance or stabilisation of carbonic anhydrase to physiologically young levels.

## MATERIALS AND METHODS

### Mitochondrial preparations

Brain, skeletal muscle and retinal tissue were dissected from young (4-11 week old) and old (78 weeks) C57BL/6J mice (Charles River). The Purkinje cell degeneration mice (*pcd^5J^*) and wild type animals (9-17 weeks old) were sourced from the University of Nottingham.

### 2D gel analysis

Mitochondrial samples, 4-11 weeks brain (n=5), 78 weeks brain (n=5), 4-11 weeks skeletal muscle (n=3) and 78 weeks skeletal muscle (n=3), were subject to iso-electric focussing using ZOOM IPG (Life Technologies) system and pH 3-10 (non-linear) ZOOM IPG strips following the manufacturers protocol. Gels were stained (SimplyBlue™ SafeStain, Life Technologies) and imaged (ImageQuant 300, GE Healthcare Life Sciences). Analysis was performed using SameSpots software (Totallab). Protein spots with a *p value* of less than 0.15 and a fold change greater than 1.2 were further analysed (one-way ANOVA). Proteins were identified from the gel pieces as described previously [5].

### Western blotting

Western blotting was carried out as described previously [5].

Antibody dilutions: Carbonic anhydrase II ab6621 (Abcam) 1:7000 dilution in 3% (w/v) BSA in TBS-T; NADH dehydrogenase flavoprotein 2 ARP57510-PO50 (Cambridge Bioscience) 1:5000 dilution in 3% (w/v) BSA in TBS-T; Beta-actin ab8227 (Abcam) 1:5000 dilution in 3% (w/v) BSA in TBS-T; Carbonic anhydrase III AP7633a (ABGENT) 1:2500 dilution in 3% (w/v) BSA in TBS-T, GAPDH G9545 (SIGMA) 1:5000 dilution in 3% (w/v) BSA in TBS-T and COXIV (ab16056) 1:5000 dilution in 3% (w/v) BSA in TBS-T. Brain mitochondrial samples were normalised to beta-actin level. The average of four samples for each condition (old and young) were plotted showing the mean +/− SEM. The muscle mitochondrial samples were normalised to GAPDH level. The average of the four samples for each condition (old and young) were plotted showing the mean +/− SEM. The retina mitochondrial samples were normalised to COXIV level. The average of the six samples for each condition (young and old) were plotted showing the mean +/− SEM. Statistical analyses (unpaired *t*-tests with Welch's correction) were carried out in GraphPad Prism.

### Carbonic anhydrase II enzyme assay

Esterase activity of carbonic anhydrase II was measured by monitoring the release of 4-nitrophenol at A348 nm in a Thermo Scientific Helios Epsilon spectrophotometer using standard methods [46]. Cuvettes contained 900 μl of 15 mM Tris Sulphate Buffer, pH 7.6 at 0°C (Sigma), 500 μl of 3 mM 4-nitrophenyl-acetate (Sigma) and 30 μg/μl of mitochondrial sample (3 mitochondrial preparations and 3 replicates for each condition, 4-11 weeks and 78 weeks). The rate of change in absorbance of the assay was plotted. Unpaired *t*-tests (GraphPad Prism) were carried out at each time point.

### Carbonic anhydrase II lifespan study

CB5600 *C. elegans* strain is superficially wild type and expresses GFP in nuclei and mitochondria of body-wall muscles. Animals were maintained on solid NGM agar plates seeded with the *Escherichia coli* strain OP50 using standard methods and aged-synchronized [47, 48]. Animals were exposed to three concentrations of synthetic carbonic anhydrase II (C2522 SIGMA) 1500 units, 150 units and 15 units. Control plates had 20 μl of dH_2_0 spotted on to the surface whilst treated plates had 20 μl of carbonic anhydrase II spotted on. Each plate contained 10 L1 larvae (n=40). Animals were scored every day and those not moving that did not respond to stimulation with a needle were recorded as dead. The experiment was maintained at 20°C. Kaplan-Meier survival curve and statistical analysis (log-rank, Mantel-Cox, test) was performed using GraphPad Prism.

### Ethical approval

Animals were bred and housed in accordance with strict Home Office stipulated conditions. The overall programme of work (in respect to the original UK Home Office Project Licence application) is reviewed by the Animal Welfare and Ethical Review Body at the University of Nottingham and then scrutinised by the UK Home Office Inspectorate before approval by the Secretary of State. Individual study protocols link to the overarching Home Office Project Licence and are made available to the Named Animal Care and Welfare Officer, the Named Veterinary Surgeon (both are members of the AWERB), the animal care staff and the research group. The Project Licence Number for the breeding and maintenance of this genetically altered line of mice is PPL 40/3576. The mice are typically group housed and maintained within solid floor cages containing bedding and nesting material with additional environmental enrichment including chew blocks and hiding tubes. Cages are Individually Ventilated Cage Units within a barrier SPF unit to maintain bio-security. Animals are checked daily by a competent and trained animal technician. Any animal giving cause for concern such as subdued behaviour, staring coat, loss of weight or loss of condition will be humanely killed using a Home Office approved Schedule 1 method of killing.

## SUPPLEMENTARY MATERIALS FIGURES


