# Supplementary material for: Mitochondrial proteomic profiling reveals increased carbonic anhydrase II in aging and neurodegeneration
Source: Aging (Albany NY). 2016 Oct 10;8(10):2425–34. doi: 10.18632/aging.101064 (PMC5115898; doi:10.18632/aging.101064)
Supplement: Supplementary file 1 [file aging-08-2425-s001.pdf]

SUPPLEMENTARY MATERIAL

A)

1	MRATDRMGAR	AVSELRLALL	FVLVLGTPRL	GVQGEDGLDF	PEYDGVDRVI
51	NVNAKNYKNV	FKKYEVLALL	YHEPPEDDKA	SQRQFEMEEL	ILELAAQVLE
101	DKGVGFGLVD	SEKDAAVAKK	LGLTEEDSVY	VFKGDEVIEY	DGEFSADTLV
151	EFLLDVLEDP	VELIEGEREL	QAFENIEDEI	KLIGYFKSKD	SEHYKAYEDA
201	AEEFHPYIPF	FATFDSKVAK	KLTLKLNEID	FYEAFMEEPM	TIPDKPNSEE
251	EIVSFVEEHR	RSTLRKLKPE	SMYETWEDDL	DGIHIVAFAE	EADPDGYEFL
301	ETLKAVAQDN	TENPDLSIIW	IDPDDFPLLV	PYWEKTFDID	LSAPQIGVVN
351	VTDADSIWME	MDNEEDLPSA	DELEDWLEDV	LEGEINTEDD	DDDDDDDDDD
401	DDDDD				

B)

1	MAVPPTYADL	GKSARDVFTK	GYGFGLIKLD	LKTKSENGLE	FTSSGSANTE
51	TTKVNGSLET	KYRWTEYGLT	FTEKWNTDNT	LGTEITVEDQ	LARGLKLTFD
101	SSFSPNTGKK	NAKIKTGYKR	EHINLGCDVD	FDIAGPSIRG	ALVLGYEGWL
151	AGYQMNFETS	KSRVTQSNFA	VGYKTDEFQL	HTNVNDGTEF	GGSIYQKVNK
201	KLETAVNLAW	TAGNSNTRFG	IAAKYQVDPD	ACFSAKVNNS	SLIGLGYTQT
251	LKPGIKLTLS	ALLDGKNVNA	GGHKLGLGLE	FQA	

C)

1	MAAPAASGLS	RQVRSFSTSV	VRPFAKLVRP	PVQVYGIEGR	YATALYSAAS
51	KEKKLDQVEK	ELLRVGQLLK	DPKVSLAVLN	PYIKRTVKVK	SLNDITKREK
101	FSPLTANLMN	LLAENGRLGN	TQGIISAFST	IMSVHRGEVP	CTVTTASPLD
151	DAVLSELKTV	LKSFLSPNQI	LKLEIKTDPS	IMGGMIVRIG	EKYVDMSAKS
201	KIQKLSKAMR	EML			

D)

1	MASKRALVIL	AKGAEEMETV	IPVDVMRRAG	IKVTVAGLAG	KDPVQCSRDV
51	MICPDTSLED	AKTQGPYDVV	VLPGGNLGAQ	NLSESPMVKE	ILKEQESRKG
101	LIAAICAGPT	ALLAHEVGFG	CKVTTHPLAK	DKMMNGSHYS	YSESRVEKDG
151	LILTSRGPGT	SFEFALAIVE	ALVGKDMANQ	VKAPLVLKD	

E)

1	MAKEWGYASH	NGPDHWHELY	PIAKGDNQSP	IELHTKDIKH	DPSLQPWSAS
51	YDPGSAKTIL	NNGKTCRVVF	DDTYDRSMLR	GGPLSGPYRL	RQFHLHWGSS
101	DDHGSEHTVD	GVKYAAELHL	VHWNPKYNTF	GEALKQPDGI	AVVGIFLKIG
151	REKGEFQILL	DALDKIKTKG	KEAPFTHFDP	SCLFPACRDY	WTYHGSFTTP
201	PCEECIVWLL	LKEPMTVSSD	QMAKLRSLFS	SAENEPPVPL	VGNWRPPQPV
251	KGRVVRASFK				

F)

1	MVLSADDKAN	IKATWEKIGG	HGAEYGAEAL	ERMFASFPTT	KTYFPHFDVS
51	HGSAQVKSHG	KKVADALANA	AHHLDDLPGA	LSALSDLHAH	KLRVDPVNFK
101	LLGHCLLVTL	ATHLQAGLTP	AAHASLDKFL	ASVSTVLTSK	YR

Supplementary Figure 1. (**A**) The peptides identified from spot 6 matched the peptide sequence of the protein calsequestrin. The peptides found in spot 6 are highlighted in red and cover 9.5% of the peptide sequence for calsequestrin. (**B**) The peptides in spot 26 matched the peptide sequence of voltage-dependent anion-selective channel protein 1 (VDAC 1). The peptides found in spot 26 are highlighted in red and covers 26% of VDAC 1 peptide sequence. (**C**) The peptides in spot 47 matched the peptide sequence of ATP synthase subunit O. The peptides found in spot 47 are highlighted in red and covers 44% of ATP synthase subunit O peptide sequence. (**D**) The peptides in spot 47 also matched the peptide sequence of protein DJ-1. The peptides found in spot 47 are highlighted in red and covers 24% of protein DJ-1 peptide sequence. (**E**) The peptides in spot 29 matched the peptide sequence of protein carbonic anhydrase III. The peptides found in spot 47 are highlighted in red and covers 19% of protein carbonic anhydrase III peptide sequence. F) The peptides in spot 60 matched the peptide sequence of protein haemoglobin subunit alpha. The peptides found in spot 60 are highlighted in red and covers 21% of protein haemoglobin subunit alpha peptide sequence.

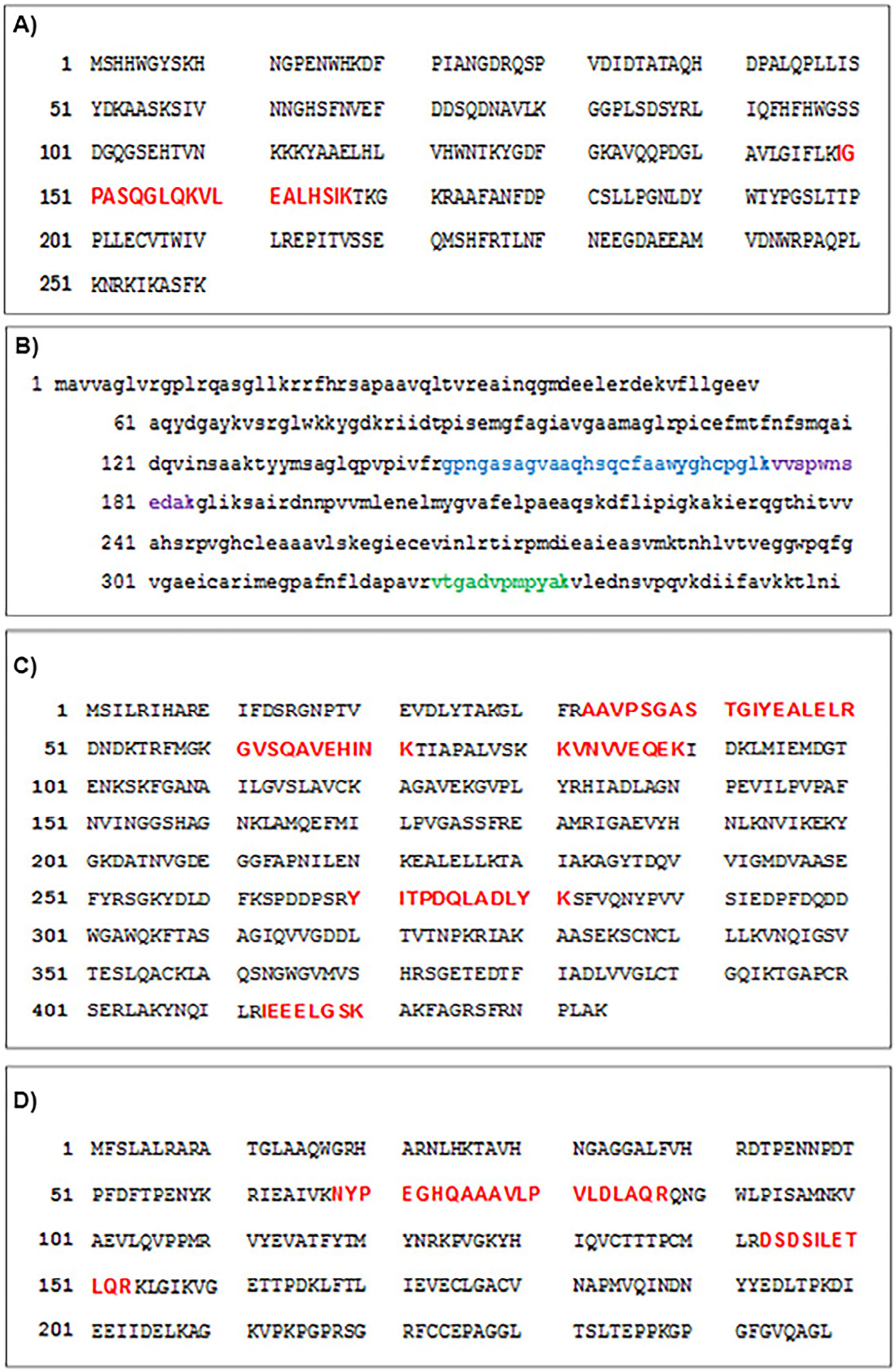

Supplementary Figure 2. (**A**) The peptides in spot 133 matched the peptide sequence of carbonic anhydrase II. The peptides found in spot 133 are highlighted in red and covers 7% of carbonic anhydrase II peptide sequence. Carbonic anhydrase II was found to increase in abundance in the aged brain mitochondrial proteome. (**B**) In addition to using the MASCOT database peptide de novo sequencing was also used to identify protein spot 108. The peptides identified in protein spot 108 matched 14% of the sequence for the protein pyruvate dehydrogenase E1 subunit β (matching peptides are highlighted in blue, purple and green). (**C**) The peptides in spot 75 matched the peptide sequence of alpha enolase. The peptides found in spot 75 are highlighted in red and covers 13% of alpha enolase peptide sequence. Alpha enolase was found to increase in abundance in the aged mitochondrial proteome. (**D**) The peptides in spot 153 matched the peptide sequence of NADH dehydrogenase flavoprotein 2. The peptides found in spot 153 are highlighted in red and covers 12% of alpha enolase peptide sequence. NADH dehydrogenase flavoprotein 2 was found to increase in abundance in the aged mitochondrial proteome.
